# The Effect of Subcutaneous and Intraperitoneal Instillation of Local Anesthetics on Postoperative Pain after Laparoscopic Varicocelectomy: A Randomized Controlled Trial

**DOI:** 10.3390/children8111051

**Published:** 2021-11-13

**Authors:** Zenon Pogorelić, Tea Gaberc, Miro Jukić, Goran Tintor, Ana Nevešćanin Biliškov, Ivana Mrklić, Ana Jerončić

**Affiliations:** 1 Department of Pediatric Surgery, University Hospital of Split, 21 000 Split, Croatia; mirojukic.mefst@gmail.com; 2 Department of Surgery, School of Medicine, University of Split, 21 000 Split, Croatia; teagaberc@gmail.com; 3 Department for Plastic, Reconstructive, Aesthetic and Reconstructive Surgery, University Hospital of Split, 21 000 Split, Croatia; gogitintor@gmail.com; 4 Department of Anesthesiology, Reanimatology and Intensive Care, University Hospital of Split, 21 000 Split, Croatia; anevescanin@gmail.com; 5 Department of Pathology, Forensic Medicine and Cytology, University Hospital of Split, 21 000 Split, Croatia; ivana.mrklic@gmail.com; 6 Department of Research in Biomedicine and Health, School of Medicine, University of Split, 21 000 Split, Croatia; ajeronci@mefst.hr

**Keywords:** local anesthetic, pain, varicocele, adolescents, laparoscopy

## Abstract

Background: The main goal of the present randomized clinical trial was to investigate the effects of subcutaneous administration of two different local anesthetics at trocar incision sites at the abdominal wall in combination with intraoperative intraperitoneal instillation of local anesthetics, on the character of postoperative pain, in adolescents who underwent laparoscopic varicocelectomy. Methods: A total of 60 patients with a median age of 16 years, who received laparoscopic varicocele repair, were included in this randomized clinical trial. The patients were randomly assigned to three study groups receiving 2% lidocaine, 0.5% levobupivacaine, or the control group. The Visual Analogue Scale (VAS) was used by a blinded nurse at four different time points (2, 6, 12 and 24 h after the surgery) to measure pain intensity. Results: The significant effect of time on the pain intensity (*p* = 0.001) was found. Additionally, the interaction between time and different local analgesics (*p* < 0.001) was observed. In patients in whom 0.5% levobupivacaine has been used, significantly lower VAS pain scores were recorded at each time point assessed, in comparison with the patients who received 2% lidocaine or the patients from the control group in whom no local anesthetic was applied (*p* < 0.001). Furthermore, in patients in whom 2% lidocaine was administrated, significantly lower pain levels according to VAS were reported than in those from the control group, except for the time point at 24 h after surgery when pain levels were comparable. Concerning the postoperative pain control, the number of patients who requested oral analgesics postoperatively was significantly lower in the group of patients in whom local anesthetic was administrated intraoperatively (2% lidocaine—n = 4, 20%; 0.5% levobupivacaine—n = 1, 5%) compared to the patients who did not receive any local anesthetic during the surgery (n = 13; 65%) (*p* < 0.001). Conclusion: A significant reduction in postoperative pain intensity and analgesics consumption in patients undergoing laparoscopic varicocelectomy who received intraoperative local anesthetic was observed. The best effect on postoperative pain intensity, according to the VAS score, was achieved by 0.5% levobupivacaine.

## 1. Introduction

A varicocele represents an abnormal enlargement of the veins within the pampiniform plexus and has been recognized as one of the most frequent causes of infertility in men, with an incidence in the pediatric population of 10–15% [1]. Around 40% of adult males with this abnormality are infertile [1,2,3]. The usual modality of treatment, with a very high success rate, is surgical varicocelectomy. Today, several surgical approaches for successful varicocele treatment are available, including open inguinal, subinguinal microscopic laparoscopic, and robotic-assisted laparoscopic ligation [1,2,3,4]. Due to the benefits of minimally invasive surgery, laparoscopic varicocelectomy has been popularized and has gained growing acceptance among pediatric surgeons [4].

Although it has been shown that pain after laparoscopic surgery is significantly less compared to open surgery, it is still present at a lower intensity [2,5,6]. For the reduction of postoperative pain following laparoscopic surgery, several interventions have been studied. One of the possibilities is the installation of local anesthetics subcutaneously under the skin incisions and intraperitoneal anesthetics administration at the site of the peritoneal defect or continuous intravenous perioperative infusion of local anesthetic [7,8]. Significant to moderate reduction of postoperative pain during ambulation has been reported after instillation of the local anesthetics at the trocar sites, while reducing the intensity of abdominal pain has been reported after intraperitoneal administration of anesthetics [7,8,9,10]. A systematic review with meta-analysis has shown that instillation of local anesthetic at the trocar site before the incision has been made or at the end of the procedure significantly reduces postoperative pain compared with placebo [11]. A more significant effect on postoperative pain control after laparoscopic surgery has been reported after intraperitoneal instillation of local anesthetics [8,11,12]. 

Since most of the published reports are among the adult population, and because the fact that the combination of incisional site and intraperitoneal analgesia has rarely been studied, the main goal of the present randomized clinical trial was to investigate the effects of subcutaneous administration of two different local anesthetics at trocar incision sites at the abdominal wall in combination with intraoperative intraperitoneal instillation of local anesthetics, on the character of postoperative pain in adolescents who underwent laparoscopic varicocele repair.

## 2. Materials and Methods

### 2.1. Patients

All pediatric patients with varicocele who underwent laparoscopic varicocelectomy and whose parents or legal guardians agreed to participation (n = 60) were enrolled in a prospective randomized study. The study was conducted from 1 May 2019 to 17 May 2021 at the Department of Pediatric Surgery, University Hospital of Split. The inclusion criteria were otherwise healthy patients, 12–18 years of age, who underwent laparoscopic varicocelectomy. The patients who received open or microsurgical varicocelectomy, those who underwent conversion to open procedure, and patients with chronic, metabolic, endocrine diseases or systemic infections were excluded. 

### 2.2. Outcomes of the Study

The primary outcome of the study was the intensity of postoperative pain. Secondary outcomes were the incidence of postoperative usage of local analgesics, the incidence of postoperative complications, and the duration of hospital stay.

### 2.3. Study Design

**Figure 1:**
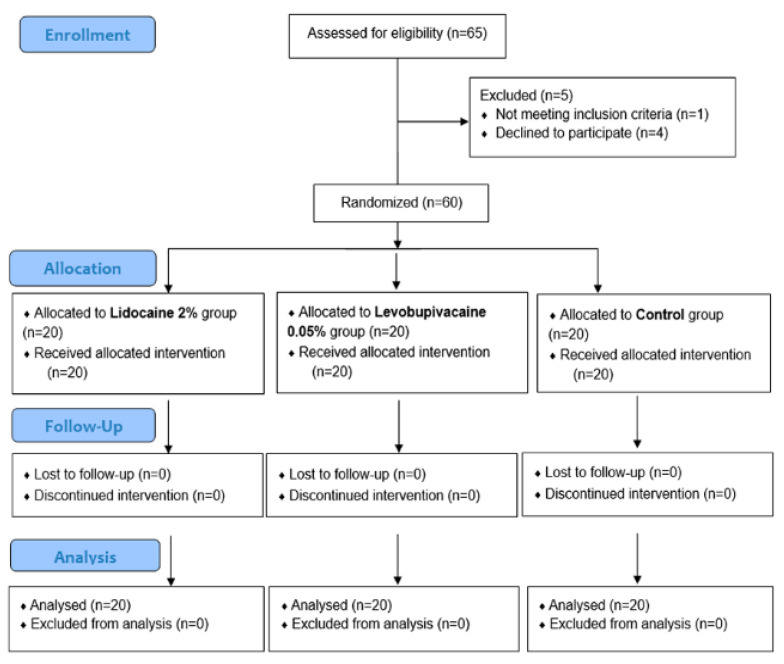
Flow chart of the study.

A total of 60 patients were randomly assigned to the study. Depending on intraoperatively administrated local analgesic, the patients were divided into three study groups (20 patients per group). In the first group of the patients, the total amount of 6 mL of 2% lidocaine (Lidokain^®^, Belupo, Koprivnica, Croatia) was injected at the three trocar insertion sites (2 mL at each) prior to incision, and 10 mL of 2% lidocaine was injected at the end of the procedure, under the direct visualization of the laparoscope, below and around the defect of the peritoneum at the site of varicocele. In the second group of the patients’ total amount of 6 mL of 0.5% levobupivacaine (Chirocaine^®^, Abbott Scandinavia AB, Solna, Sweden) was injected at the three trocar insertion sites (2 mL at each) prior to incision and 10 mL of 0.5% levobupivacaine was injected at the end of the procedure, under the direct visualization of the laparoscope, below and around the defect of the peritoneum at the site of varicocele. The third group was the control group; there was no local or peritoneal administration of any local anesthetic prior, during, and after the surgical procedure. The Visual Analogue Scale (VAS) was used to measure pain intensity [13]. The VAS (0–10 scale) pain score was measured by a blinded nurse at four different time points (2, 6, 12 and 24 h after the surgery). The study protocol envisaged collection of the following data: demographic data (age, weight, height, and body mass index), characteristics of varicocele (lateralization, diameter of veins, and indication for surgery), intraoperative data (intraoperative finding, duration of surgery, and intraoperative complications) and postoperative follow-up (length of hospital stay, postoperative complications, VAS score at 2, 6, 12 and 24 h after surgery, and usage of analgesics). The flow chart diagram of the study is shown in Figure 1.

### 2.4. Compliance with Ethical Standards

Prior to being included in the study, the patients were informed in detail about the procedure and possible risks and complications. Informed consent to use the data was obtained from the patients’ parents or their legal guardians. The study protocol was approved by the Ethics Review Board of the University hospital of Split (reference: 500-03/20-01/09; date of approval: 1 June 2020). The study was registered in the ClinicalTrials.gov registry under the identifier NCT05034406.

### 2.5. Operative Technique and Anesthesia

The same anesthesiologist performed all procedures under general anesthesia. Induction of anesthesia was performed using fentanyl (Piramal, West Drayton, UK) (4 μg/kg), propofol (Propofol, Fresenius Kabi Austria GmbH^©^, Linz, Austria) (4 mg/kg), and vecuronium bromide (Vecurol, Demo S.A., Thermi, Greece) (0.05 mg/kg). After 20 s, the laryngeal mask was inserted [14]. Anesthesia was maintained using air/oxygen (50%/50%) and infusion of propofol followed by Mc Farlan [15]. Paracetamol (Perfalgan, Bristol-Myers Squibb Pharmaceuticals Limited, Bristol, UK) (15 mg/kg) was administered at the end of the procedure as postoperative analgesia. 

**Figure 2:**
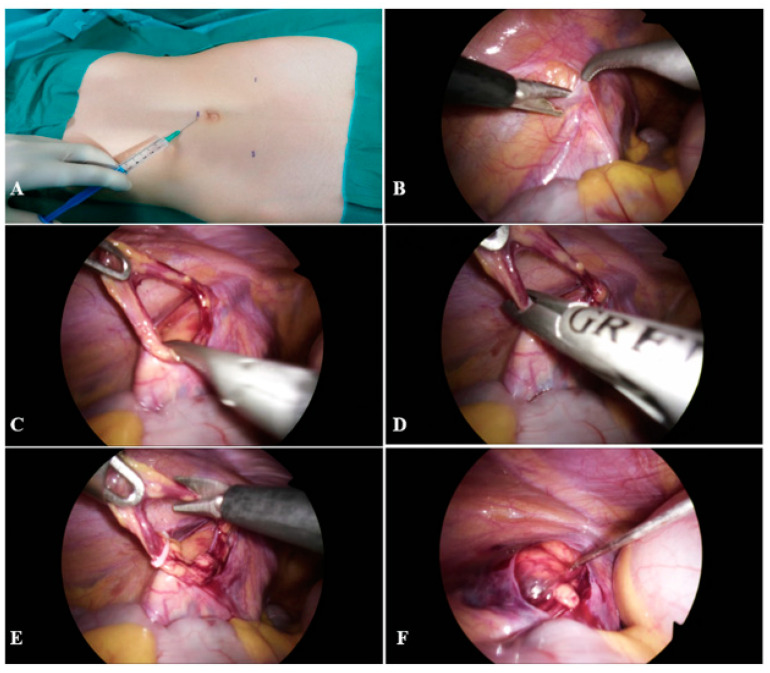
Laparoscopic varicocelectomy: (**A**)—Subcutaneous instillation of local anesthetic at trocar sites; (**B**)—Identification of spermatic vessels and opening of peritoneum using laparoscopic scissors; (**C**)—Spermatic blood vessels preparation; (**D**)—Placement of nonabsorbable polymeric ligating clips on the spermatic blood vessels; (**E**)—Resection of spermatic blood vessels; (**F**)—Instillation of local anesthetic below and around the site of the peritoneal defect.

A 2 mL of local anesthetic was injected subcutaneously in the supraumbilical region. After 30 s supraumbilical skin incision, 5 mm in length, was performed. Through the supraumbilical incision, a Veress needle was introduced, and CO_2_ pneumoperitoneum was established. After achieving the pneumoperitoneum, first 5 mm trocar was introduced through the same incision. Then, a local anesthetic was injected subcutaneously in both midclavicular lines, 10–20 mm below the horizontal line to the umbilicus, along the lateral border of each abdominal rectus muscle. After 30 s, two 5 mm incisions were performed at the site of the previous injection of local anesthetic, and two additional 5 mm trocars were inserted (Figure 2A). The abdominal cavity was inspected using a 5 mm laparoscope (Olympus, Tokyo, Japan). After the spermatic vessels and vas deferens were identified, the peritoneum was opened 1–2 cm superior to the internal inguinal ring to expose the spermatic blood vessels (Figure 2B,C). After mobilization of the vessels, nonabsorbable polymeric ligating clips (Ligating Clips ML; Grena, Brentford, UK) were placed on the spermatic blood vessels, and the blood vessels were resected between the two clips (Figure 2D–E). At the end of the procedure, a local anesthetic was applied below and around the site of the peritoneal defect (Figure 2F). After controlling hemostasis, CO_2_ was eliminated from the abdominal cavity, trocars were removed, and skin incisions were sutured with nonabsorbable sutures (Surgipro™ II, polypropylene 3/0, Covidien, Dublin, Ireland).

### 2.6. Randomization and Sample Size Calculation

As the highest level of pain is expected 6 h after the surgery, a power calculation was performed for this time point. The expected effect size f of 2.34 was estimated from scores on the VAS scale that were observed within a pilot study, including three study groups and 45 participants in total. Under the assumption of 95% of power, a significance level of 0.05, and considering the three study groups, the minimal sample size to detect large differences with the repeated measures between-the-factors ANOVA was 9 (3 per group). Although our study was powered enough, we nevertheless enrolled 20 patients per group to allow for randomization to take effect (a study with 3 participants per group would not assure equal randomization of patients). The allocation of participants in each study group was performed by block randomization using block sizes 3 and 6. The randomization was performed by an independent statistician using computer-generated random numbers.

### 2.7. Statistical Analysis

The statistical analysis was performed using SPSS 24.0 software (IBM Corp., Armonk, NY, USA). In the case where no significant deviation from normality was observed, the mean and standard deviation was used to describe a distribution of a quantitative variable. Median and range were used for asymmetrically distributed quantitative or ordinal variables. Absolute and relative frequencies were used to describe the distribution of categorical variables. The significance of differences in quantitative variables between the three study groups was assessed with one-way ANOVA for independent samples or, an alternative, the nonparametric independent-samples Kruskal–Wallis test. The Chi-square test was used to assess differences in the distribution of categorical data. In cases when the frequency of events was low, Fisher exact test was used instead. For repeated measures on the VAS scale in four time-points (2, 6, 12, and 24 h after the surgery) general linear model for repeated measures was used. We also presented the results of repeated measurements graphically by showing mean VAS values for each time-point and each study group, with the corresponding 95% CI. All the tests were two-sided, and the significance level of 0.05 was used.

## 3. Results

A total of 60 male patients were enrolled in this randomized clinical trial. The median age of the patients was 16 (range 13–17) years. Left-sided varicocele was recorded in all study groups. The most common indication for surgery was testicular hypotrophy > 20% (n = 28 or 47%), followed by abnormal semen analysis results (n = 24 or 40%), pain (n = 15 or 25%), and bilateral varicocele (n = 1 or 2%). There was no dropout of the participants included in the study, i.e., all subjects were examined for pain intensity on the VAS scale at all time points. No differences between the study groups were found regarding demographic, clinical, and postoperative data (Table 1). The statistically significant difference between the study groups was observed only with regards to the duration of anesthesia (*p* = 0.006). No intraoperative or postoperative complications were recorded. 

General linear repeated measure model analysis revealed the significant effect of time on intensity of pain detected on the VAS scale (F = 39.27, df = 3, *p* = 0.001) as well as the interaction between time and local analgesic groups (F = 6.61, df = 6, *p* < 0.001). A significant difference was detected between the groups (F = 60.63, df = 2, *p* < 0.001) (Table 2). 

**Figure 3:**
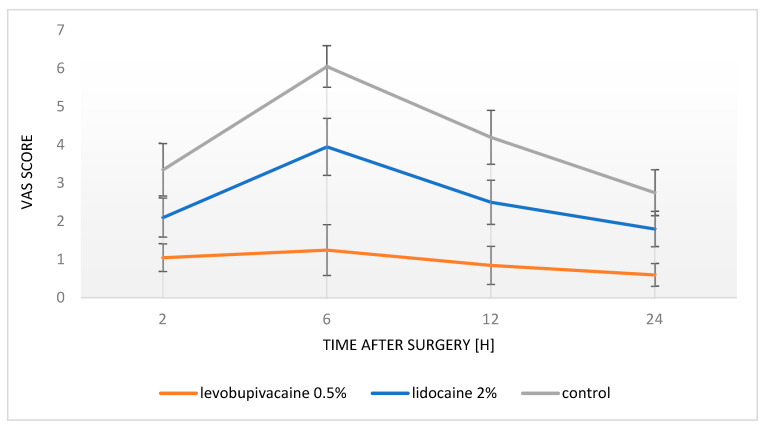
Mean values of pain intensity according to the VAS scale for every time point after surgery.

Namely, in patients in whom 0.5% levobupivacaine has been used, significantly lower VAS pain scores were recorded at each time point assessed, in comparison with the patients who received 2% lidocaine, or the patients from the control group in whom no local anesthetic was applied (*p* < 0.001) (Figure 3). 

Furthermore, the patients in whom 2% lidocaine was applied reported significantly lower pain intensity at VAS than those in the control group, except for the time point at 24 h postoperatively when pain levels were comparable. The results of repeated measurements were also presented graphically by showing mean VAS values for each time point and each study group, with the corresponding 95% CI. Concerning postoperative pain control, the number of patients who requested oral analgesics postoperatively was significantly lower in the group of patients in whom local anesthetic was administrated intraoperatively (2% lidocaine—n = 4, 20%; 0.5% levobupivacaine—n = 1, 5%) compared with the patients who did not receive any local anesthetic during the surgery (n = 13; 65%) (*p* < 0.001).

## 4. Discussion

This study demonstrated with high certainty the positive effect of local anesthesia administration on postoperative pain control in adolescents who underwent laparoscopic varicocelectomy. The best effect on postoperative pain level was achieved by 0.5% levobupivacaine administration, which resulted in the lowest intensity of pain on the VAS scale for every time point (medium 1 point on VAS scale for all time points). Furthermore, 2% lidocaine also showed a positive effect on postoperative pain control (medium difference of 2 points on VAS scale compared to control for 2, 6, and 12 h time points), but resulted in higher pain levels in comparison to adolescents who received 0.5% levobupivacaine. The adolescents who did not receive any local anesthetic intraoperatively reported significantly higher pain levels than those who received local anesthetics. Moreover, only 5–20% of patients who received local anesthetics intraoperatively required analgesics postoperatively compared with 65% of patients who did not receive local anesthetics.

Many medical professionals and patients think that laparoscopic surgery is painless. Although the level of pain after laparoscopic procedures is significantly lower than after open surgery, it has been proven in various studies that laparoscopic surgery is not entirely pain-free, especially during the early postoperative period (up to 6 h) and in some cases may require a higher amount of analgesics compared to similar open procedures [11,12]. In an attempt to reduce the level of pain in the early postoperative period after laparoscopic procedures, a variety of methods and their combinations can be performed: postoperative opioid analgesics administration either intravenously or per os, a combination of the opioids with nonsteroid anti-inflammatory drugs, local anesthetics infiltration at incision sites pre- or postoperatively, and even intraperitoneal administration of local anesthetics prior or at the end of surgery. Based on our experience and scientific research so far, all methods mentioned above are safe and useful in postoperative pain control. However, no particular method, or a combination of methods, has proven its superiority over others [8,16]. In our study, the combination of pre and postoperative administration of local anesthetics has shown to be successful in postoperative pain control in patients who underwent laparoscopic varicocelectomy. It would be interesting to conduct new research to assess the success of our findings in other kinds of laparoscopic surgeries and among patients of adult age.

Although the high incidence of varicocele varies from 14% to 20% between different studies, the incidence of varicocele among infertile men is not higher than 20% in most recently published studies [17,18,19]. The latest meta-analysis has shown that the surgical treatment of varicocele resulted in enlargement of affected testis in 40–100% cases, and the improvements of sperm density and motility have also been reported [2,18,19]. In order to prevent varicocele as the most likely cause of male infertility, patients should be treated early, preferably in adolescence, and the postoperative pain development in patients who undergo the laparoscopic surgical treatment should be prevented by local anesthetic administration intraoperatively to improve patient outcomes [9,11,16].

A recent systematic review and meta-analysis of the incision-site local analgesia showed that infiltration of local anesthetics prior to incision has no benefit over postoperative infiltration. However, when local anesthetics are administered intraperitoneally, preemptive local anesthesia decreases postoperative pain compared with the patients who received anesthesia after surgery [11]. Nevertheless, given that both incisional and intraperitoneal local analgesia reduces the level of postoperative pain, it is reasonable to question whether giving them together would result in any added benefits. In our study, this hypothesis was tested, and results showed even better effects on postoperative pain. After local anesthetic administration at the three trocar insertion sites prior to incision as well as at the end of the procedure, below and around the defect of the peritoneum at the site of varicocele, measured levels of pain were significantly lower, as well as the demand for postoperative analgesia. Chou et al. have also reported that intraperitoneal bupivacaine administration both immediately after placement of trocars and at the end of surgery was effective in reducing the intensity of pain at 2 and 4 h postoperatively [20].

Thanapal et al. evaluated the local anesthetic application at the operative site assuming that absorption may occur via the large surface of the peritoneum, further enhancing the analgesic effect of the administered local anesthetic. The study results proved that operative site-local anesthesia significantly improves postoperative pain immediately after and up to 24 h after the procedure and reduces the postoperative analgesic consumption [16]. Similar to our findings, the best results were obtained when 0.5% levobupivacaine was used.

Although Gluck et al. detected no significant differences in postoperative pain intensity among patients undergoing operative gynecologic laparoscopy who were given analgesic agents either subcutaneously or intraperitoneally, nevertheless they found that a minimum of 5 mg (20 mL of 0.25%) bupivacaine instilled subcutaneously effectively reduces postoperative pain even in diagnostic laparoscopies [8].

Custovic et al. observed patients who underwent laparoscopic appendectomy and concluded that 0.5% levobupivacaine is the more effective agent in postoperative pain control, compared with 0.5% ropivacaine, 1% lidocaine, and the control group, and also resulted in shorter hospitalization stay. According to the VAS scale, the level of pain was the lowest among patients who received 0.5% levobupivacaine and lasted up to 48 h after the procedure. They also observed a reduction in postoperative nausea and vomiting in patients who received levobupivacaine preemptively at the skin incisions and postemptively, intraperitoneally [9]. The levobupivacaine showed a significantly longer-lasting effect compared with other amide anesthetics (up to 9 h) due to its specific liposomal structure, which makes levobupivacaine a better choice than other local anesthetics in postoperative pain control [21].

The goal of the latest meta-analysis conducted by Abdelhakim et al., which included seven randomized controlled trials with 579 patients in total, was to review the success of the intraperitoneal application of the local anesthetics in patients undergoing laparoscopic appendectomy. The results of the analysis showed a significant improvement in the postoperative pain control in patients who received the intraperitoneal application of local anesthetics at different time points compared with the control group of patients. A reduced frequency of postoperative nausea and vomiting, less postoperative opioid consumption, and shorter hospital stay were also recorded in patients who received intraperitoneal local anesthetics during the surgery [22].

Nowadays, anesthesiologists have a directive to avoid the excessive use of opioids for postoperative pain treatment because of their potential life-threatening side effects, such as respiratory depression, bradycardia, and hypotension in case of opioid overdose [14,23,24]. The infiltration of local anesthetics in the surgical site does not produce any significant harm to the tissue-healing process and has the potential for reducing opioid consumption. Thus the application of local anesthetics should play an important role in controlling the pain after various surgical procedures and should be recommended, not only in laparoscopic but also in open surgeries [24,25].

A possible weakness of our study is linked to the fact that objective pain assessment is not possible since pain is solely subjective in its character. Considering that perception of pain is individual, it is almost impossible to objectively compare the results measured according to the VAS scale among our patients. Moreover, our study was conducted only in our center. Unfortunately, because of the lack of studies assessing postoperative pain in patients undergoing laparoscopic varicocelectomy, we had to compare our results to studies examining patients undergoing different laparoscopic surgeries. To implement our findings in standardized protocols for laparoscopic varicocelectomy, our results should be replicated in additional prospective multicenter studies that are fully powered, similar to our study.

## 5. Conclusions

Compared to the control group, the level of postoperative pain was significantly lower in patients who received local anesthetic intraoperatively. The best effect on postoperative pain intensity was achieved by 0.5% levobupivacaine administered at the three trocar insertion sites and the end of the procedure, below and around the defect of the peritoneum at the site of varicocele. The patients who received a local anesthetic intraoperatively required significantly less analgesia postoperatively than the patients who did not receive any local anesthesia during the surgical procedure.

## Figures and Tables

**Table 1:** Demographic, clinical and follow-up data of the patients.

Variables	Group I	Group II	Group III	*p*
	Lidocaine 2%	Levobupivacaine 0.5%	Contol	
Demographic data				
Age (years)	16	16.5	16	0.948
median (range)	(13–17)	(14–17)	(14–17)	
Height (cm)	183	184	182	0.800
median (range)	(145–196)	(168–193)	(172–189)	
Weight (kg)	71 ± 13	72 ± 10	71 ± 9	0.916
mean ± SD				
BMI (kg/m^2^)	21.5 ± 3.2	21.7 ± 2.2	21.5 ± 2.8	0.973
mean ± SD				
Clinical data				
Diameter of veins (mm)	3.8 ± 0.8	4.0 ± 0.8	3.8 ± 0.5	0.782
mean ± SD				
Lateralization, n (%)				
Left	20 (100)	18 (90)	20 (100)	0.322
Right	0 (0)	1 (5)	0 (0)	
Bilateral	0 (0)	1 (5)	0 (0)	
Indication for surgery, n (%)				
Abnormal spermiogram	8 (40)	9 (45)	9 (45)	>0.999
Testicular hypotrophy	10 (50)	10 (50)	8 (40)	0.756
Pain	5 (25)	4 (20)	6 (30)	0.930
Bilateral varicocele	0 (0)	1 (5)	0 (0)	0.322
Intraoperative and postoperative follow-up				
Duration of surgery (min)	12	13	15	0.186
median (range)	(9–25)	(9–28)	(10–30)	
Duration of anesthesia (min)	25	28	35	0.006 *
median (range)	(20–45)	(21–48)	(25–60)	
LOS (days); Median (range)	1 (1–1)	1 (1–1)	1 (1–1)	>0.999

* Significant at 0.05 level; SD—Standard deviation; BMI—Body Mass Index; LOS—Length of stay.

**Table 2:** Postoperative analgesia and pain level according to VAS.

Variables, Median (Range)	Group I	Group II	Group III	*p*
	Lidocaine 2%	Levobupivacaine 0.5%	Contol	
VAS after 2 h	2 (0–4)	1 (0–3)	4 (0–6)	<0.001 *
VAS after 6 h	4 (0–7)	1 (0–6)	6 (3–8)	
VAS after 12 h	3 (0–5)	1 (0–4)	5 (1–6)	
VAS after 24 h	2 (0–4)	1 (0–2)	3 (1–6)	
Postoperative analgesia, n (%)	4 (20%)	1 (5%)	13 (65%)	<0.001 ^†^

VAS—Visual Analog Scale; * General linear model for repeated measures; ^†^ Fisher exact test.

## Data Availability

The data presented in this study is available upon request of the respective author. Due to the protection of personal data, the data is not publicly available.
